# Supplementary material for: Comparative Evaluation of the In Vitro Cytotoxicity of a Series of Chitosans and Chitooligosaccharides Water-Soluble at Physiological pH
Source: Polymers (Basel). 2023 Sep 6;15(18):3679. doi: 10.3390/polym15183679 (PMC10537996; doi:10.3390/polym15183679)
Supplement: Supplementary file 1 [file polymers-15-03679-s001.zip › polymers-2580247-supplementary.pdf]

# Supplementary materials: Comparative Evaluation of the In Vitro Cytotoxicity of a Series of Chitosans and Chitooligosaccharides Water-Soluble at Physiological pH

**Catia Dias [1,*], Loris Commin [1], Catherine Bonnefont-Rebeix [1], Samuel Buff [1], Pierre Bruyère [1] and Stéphane Trombotto [2]**

[1] UPSP 2021.A104 ICE, Interaction Cellule Environnement, VetAgro Sup, Université de Lyon, F-69280 Marcy l'Etoile, France; loris.commin@vetagro-sup.fr (L.C.); catherine.bonnefont@vetagro-sup.fr (C.B.-R.); samuel.buff@vetagro-sup.fr (S.B.); pierre.bruyere@vetagro-sup.fr (P.B.)

[2] Univ Lyon, CNRS, UMR 5223, Ingénierie des Matériaux Polymères, Université Claude Bernard Lyon 1, INSA Lyon, Université Jean Monnet, F-69622 Villeurbanne Cédex, France ; stephane.trombotto@univ-lyon1.fr

* Correspondence: catia.silva-dias@vetagro-sup.fr; Tel.: +33-768920556

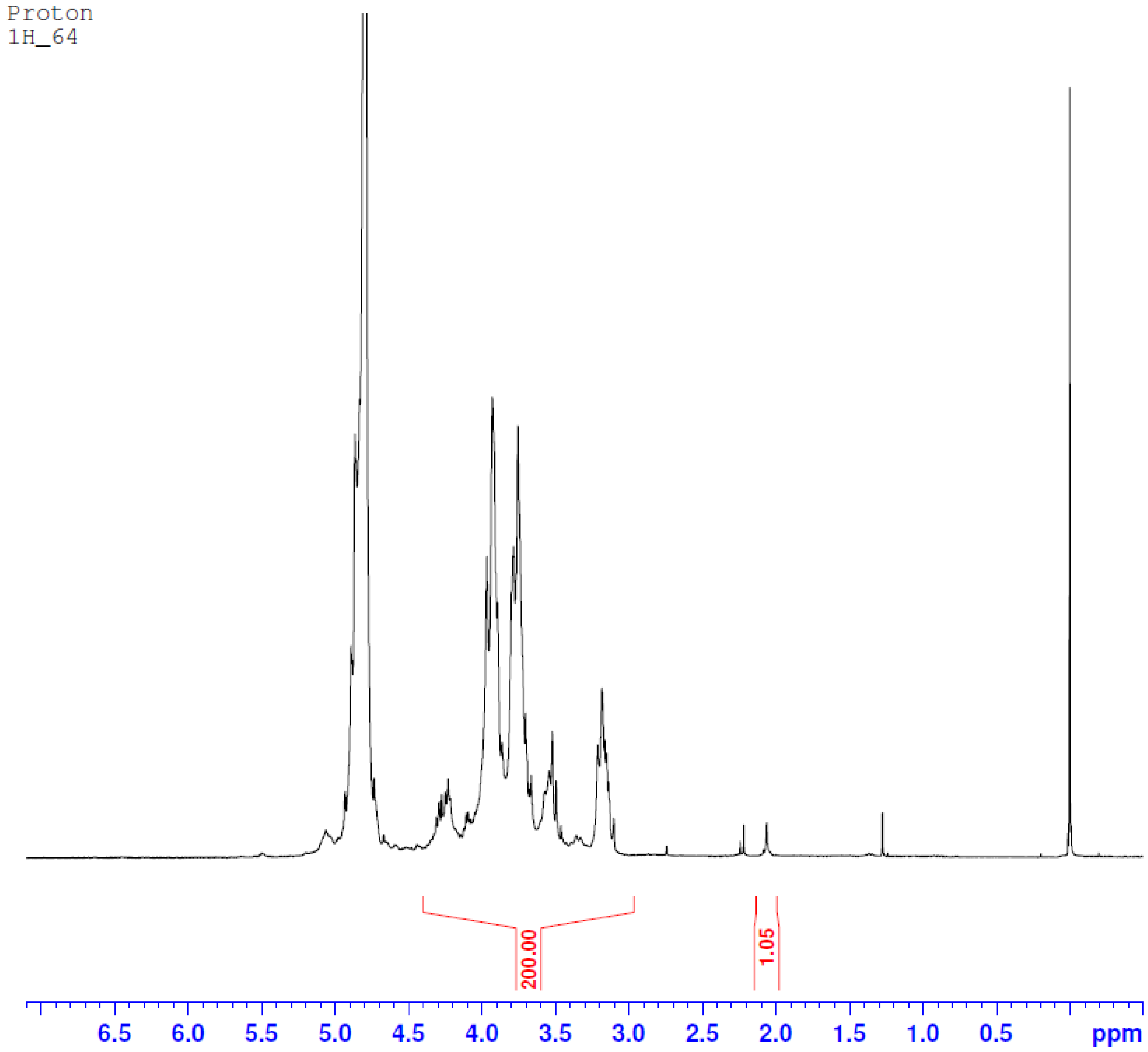


**Figure S1**. $^1$H NMR spectrum (300 MHz, 298 K) of COS$_{17/1}$ in $D_2O$.

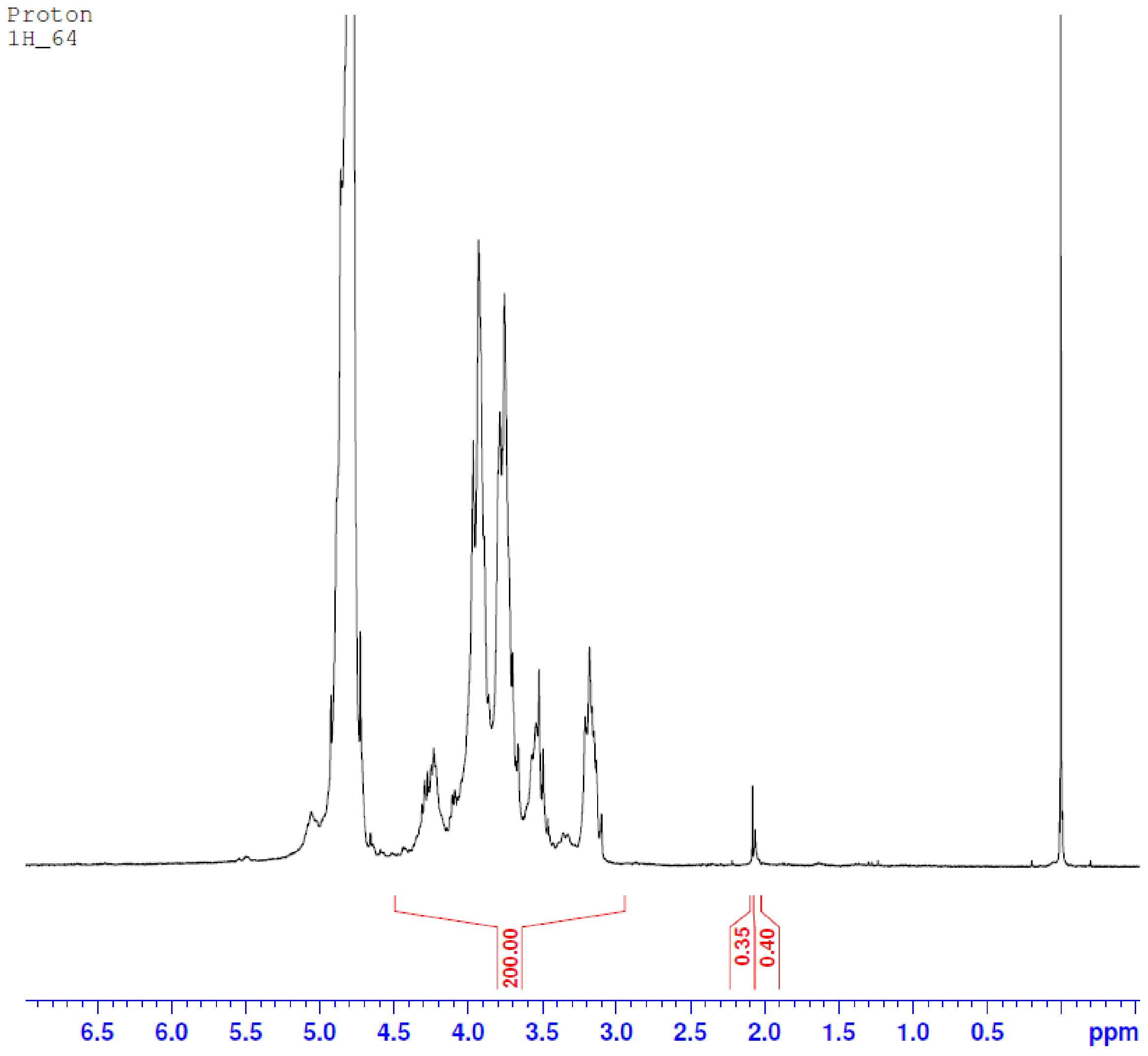


**Figure S2**. $^{1}$H NMR spectrum (300 MHz, 298 K) of $COS_{22/0}$ in $D_2O$.

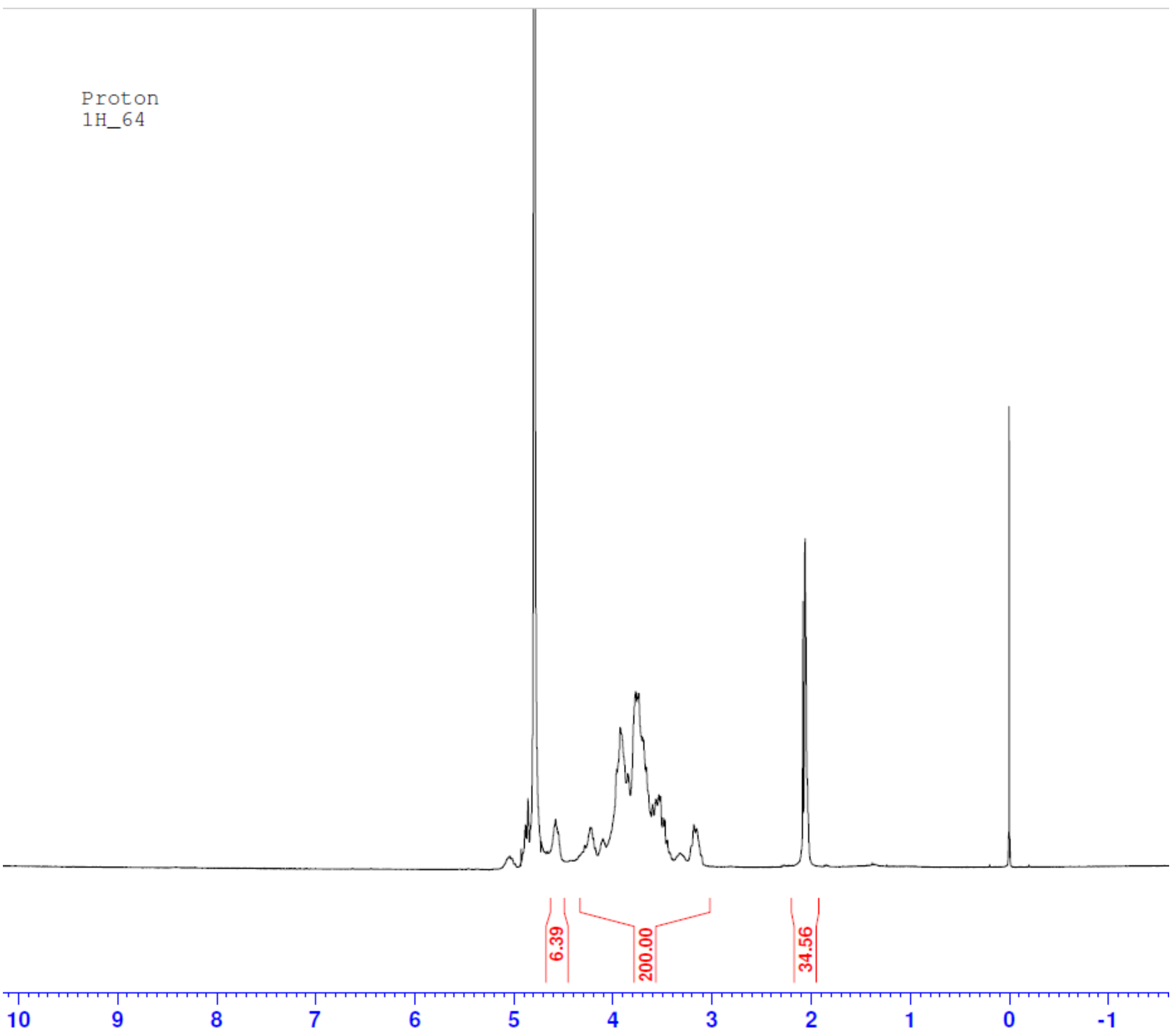


**Figure S3.** $^{1}$H NMR spectrum (300 MHz, 298 K) of $COS_{18/35}$ in $D_2O$.

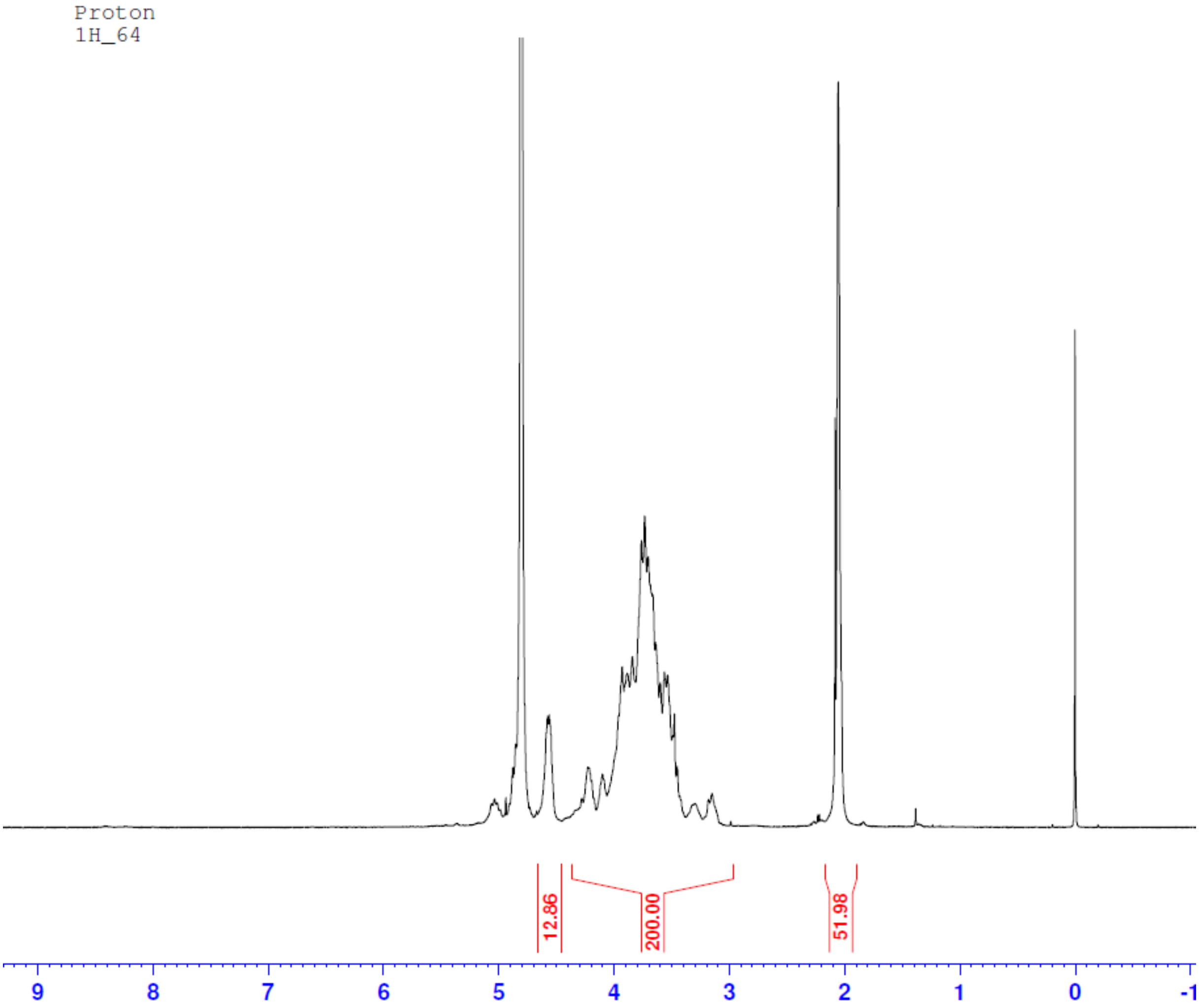


**Figure S4.** $^{1}H$ NMR spectrum (300 MHz, 298 K) of $COS_{17/51}$ in $D_2O$.

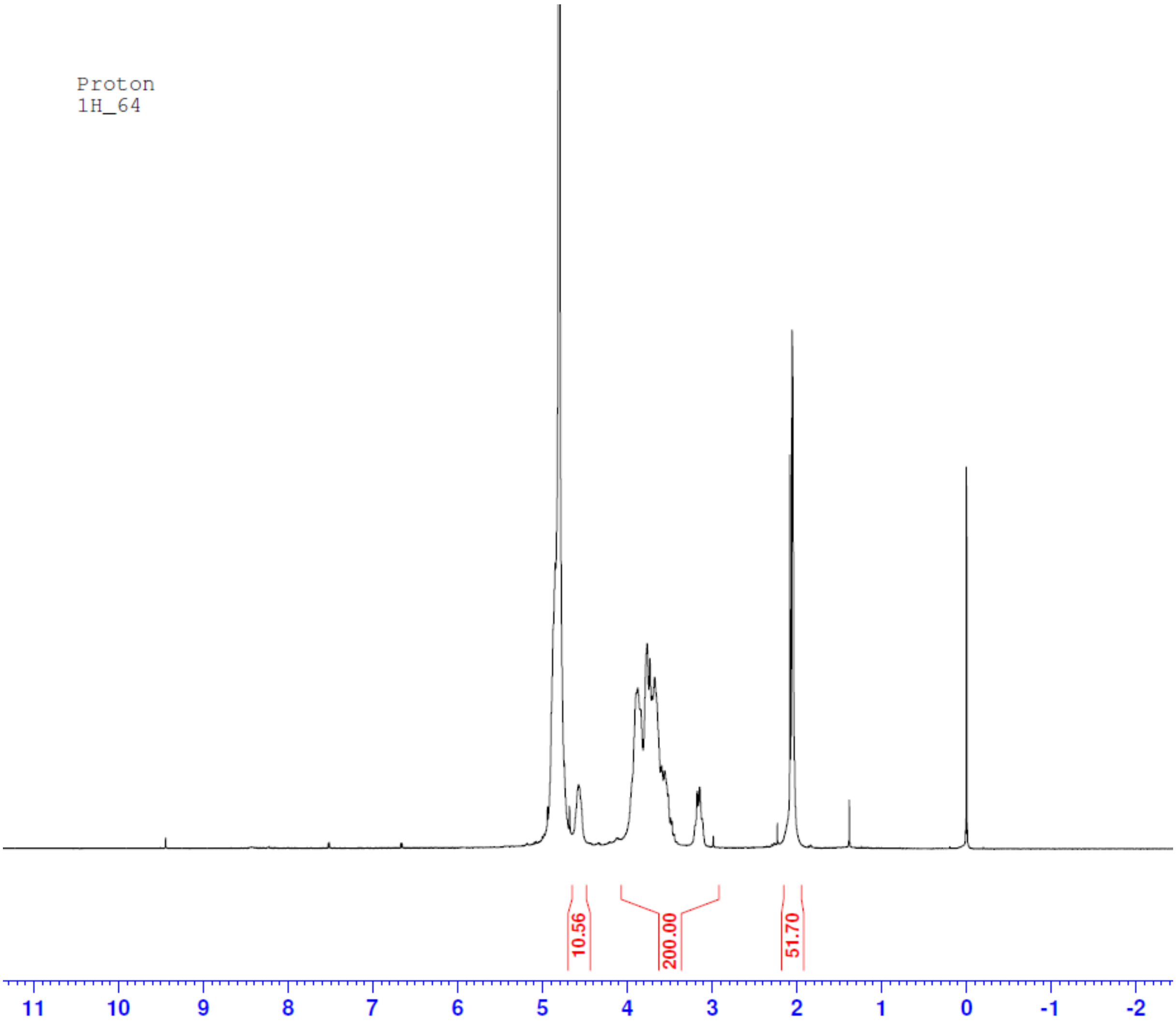


**Figure S5.** $^{1}$H NMR spectrum (300 MHz, 298 K) of $COS_{22/52}$ in $D_2O$.

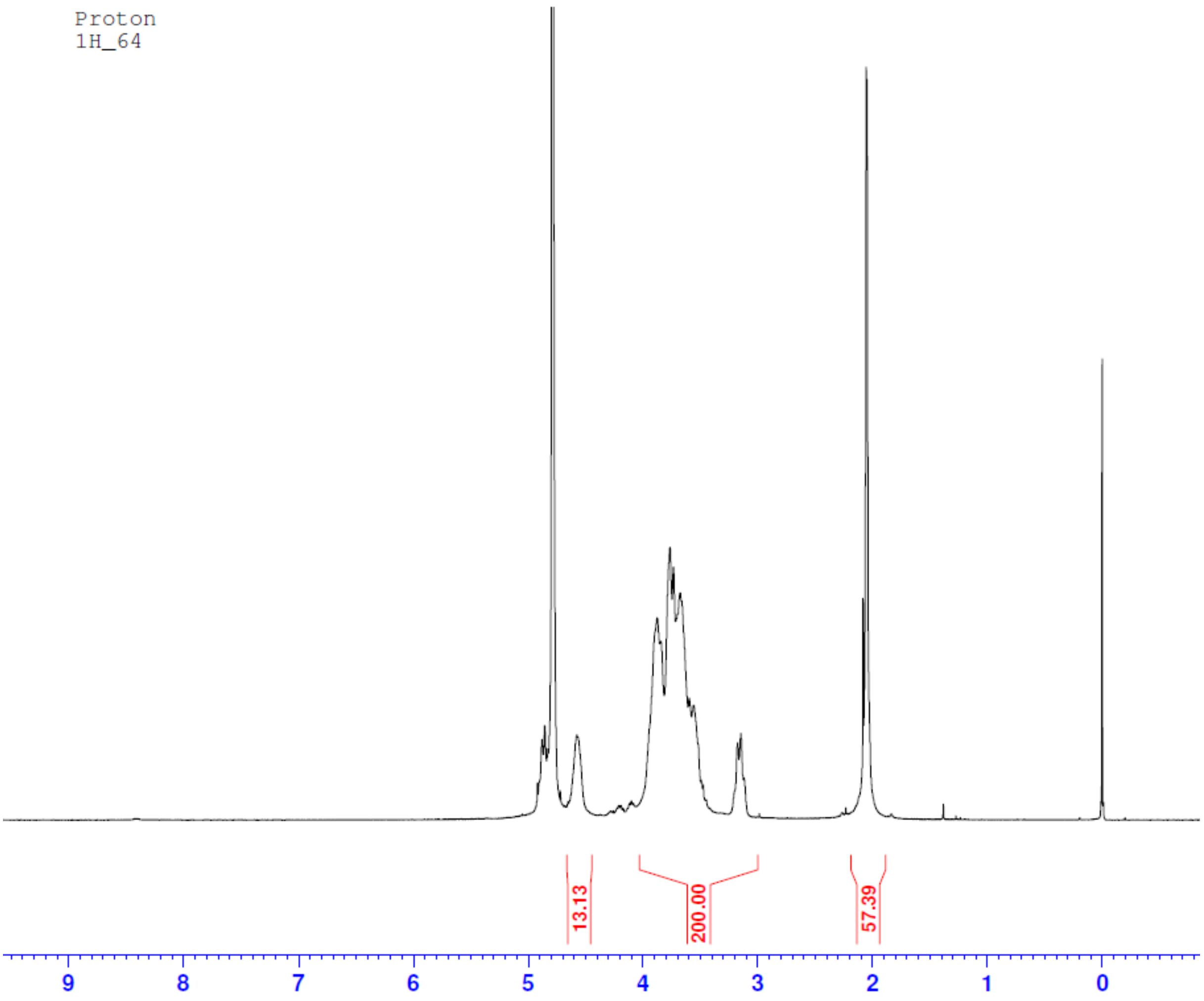


**Figure S6.** $^{1}$H NMR spectrum (300 MHz, 298 K) of $COS_{36/57}$ in $D_2O$.

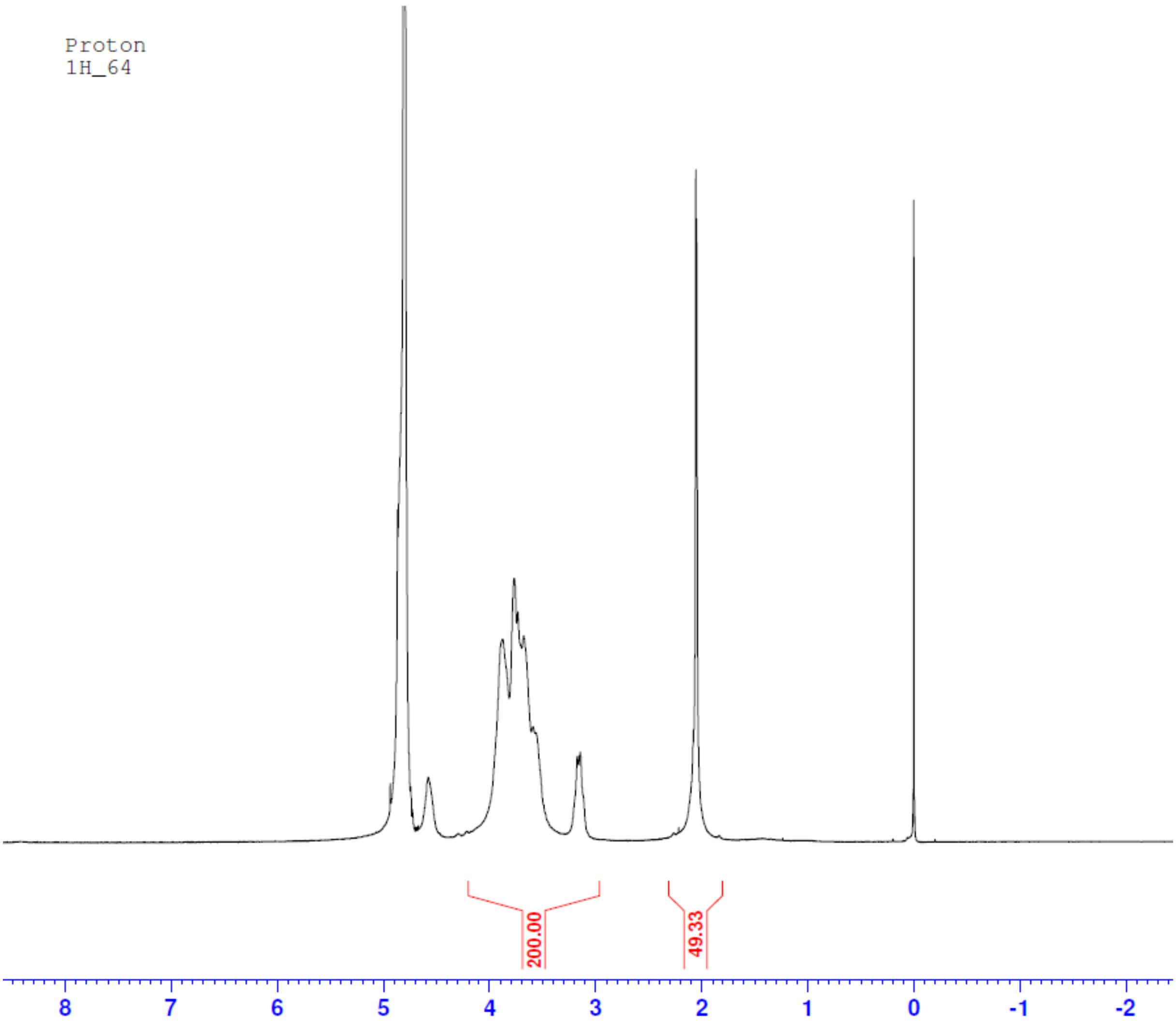


**Figure S7.** $^1H$ NMR spectrum (300 MHz, 298 K) of $CS_{100/49}$ in $D_2O$.

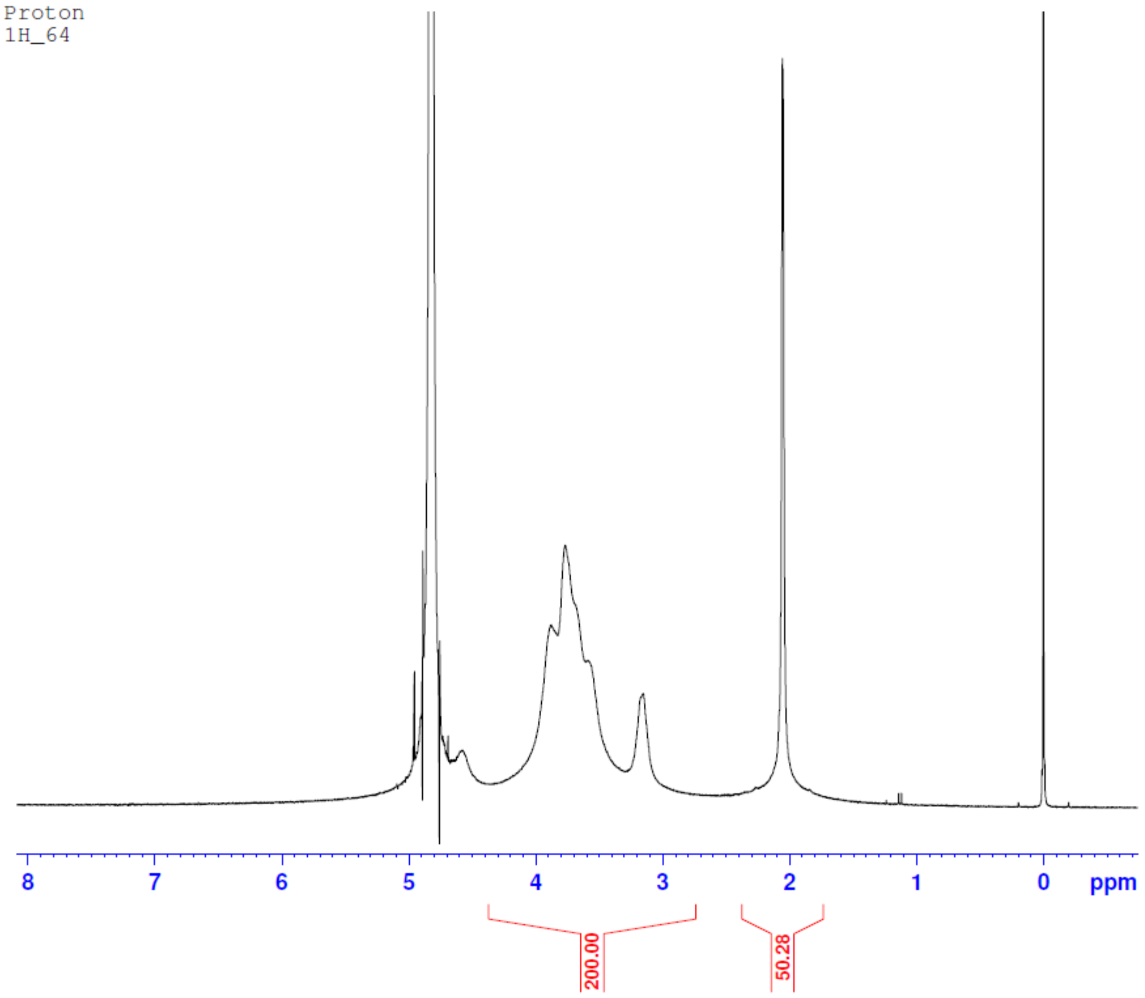


**Figure S8.** $^{1}$H NMR spectrum (300 MHz, 298 K) of $CS_{984/50}$ in $D_2O$.

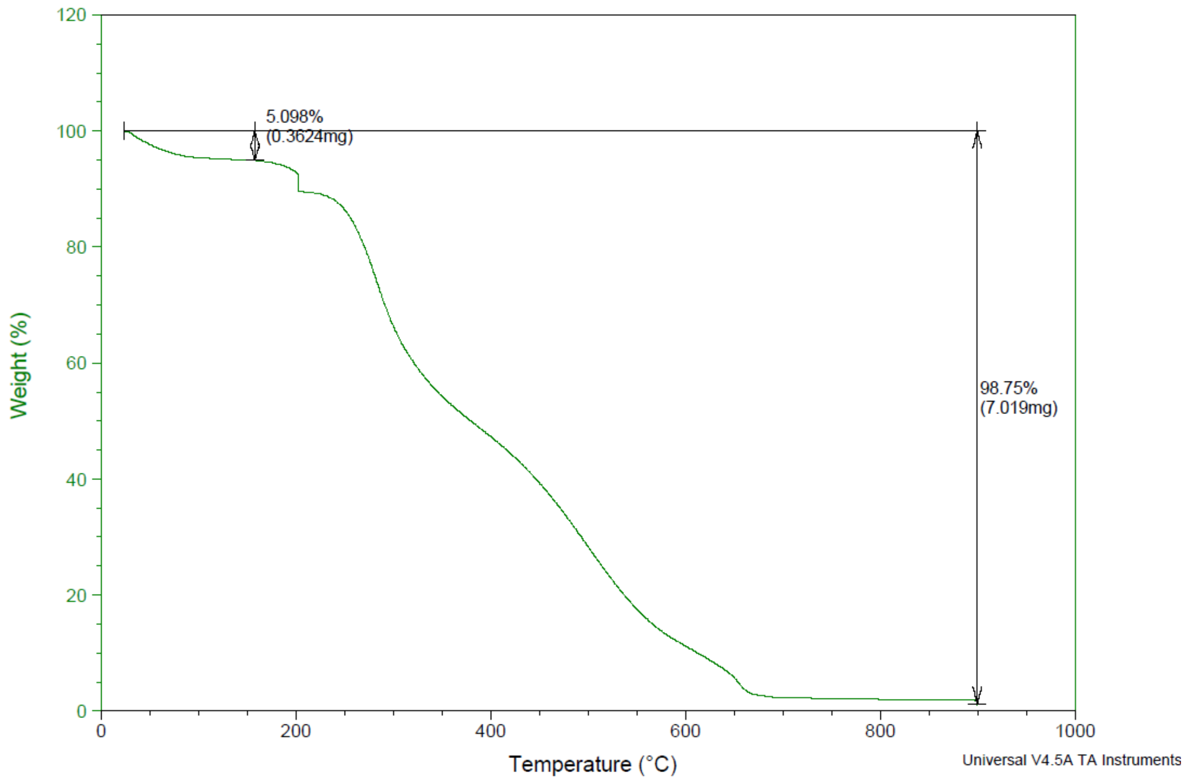


**Figure S9.** TGA thermogram of $COS_{17/1}$.

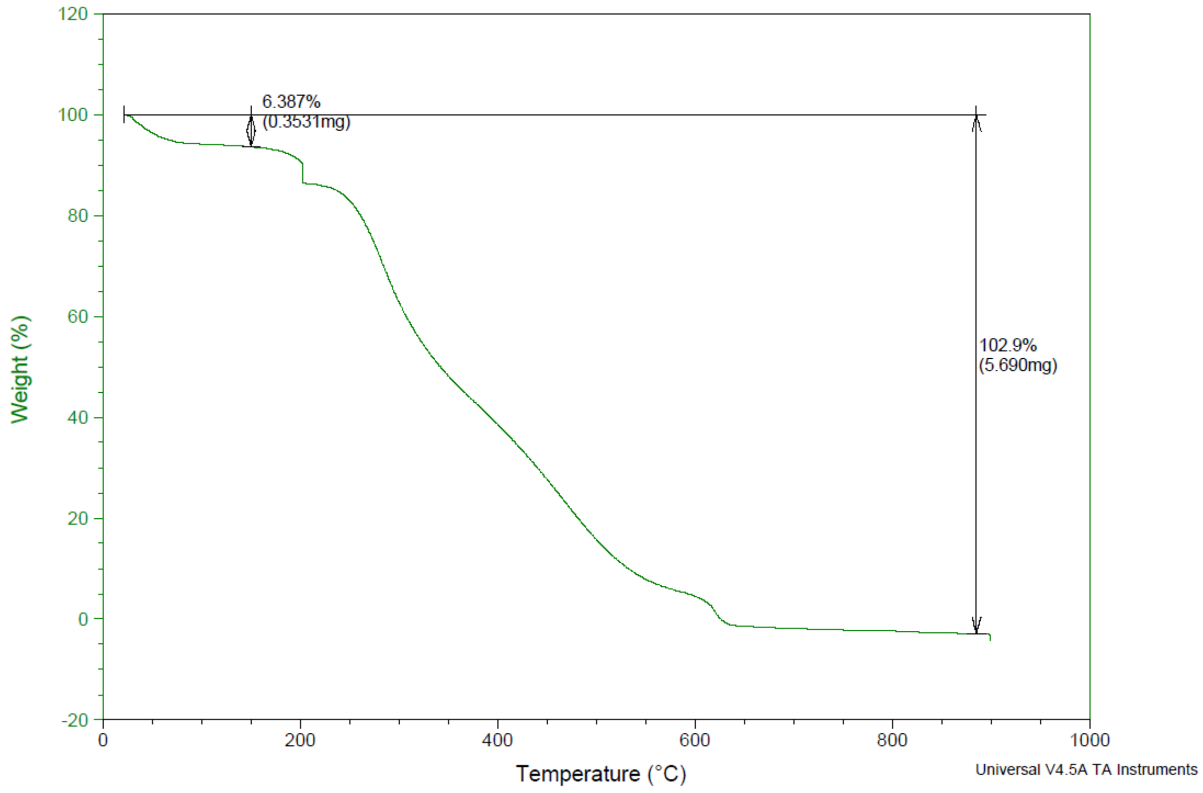


**Figure S10.** TGA thermogram of $COS_{22/0}$.

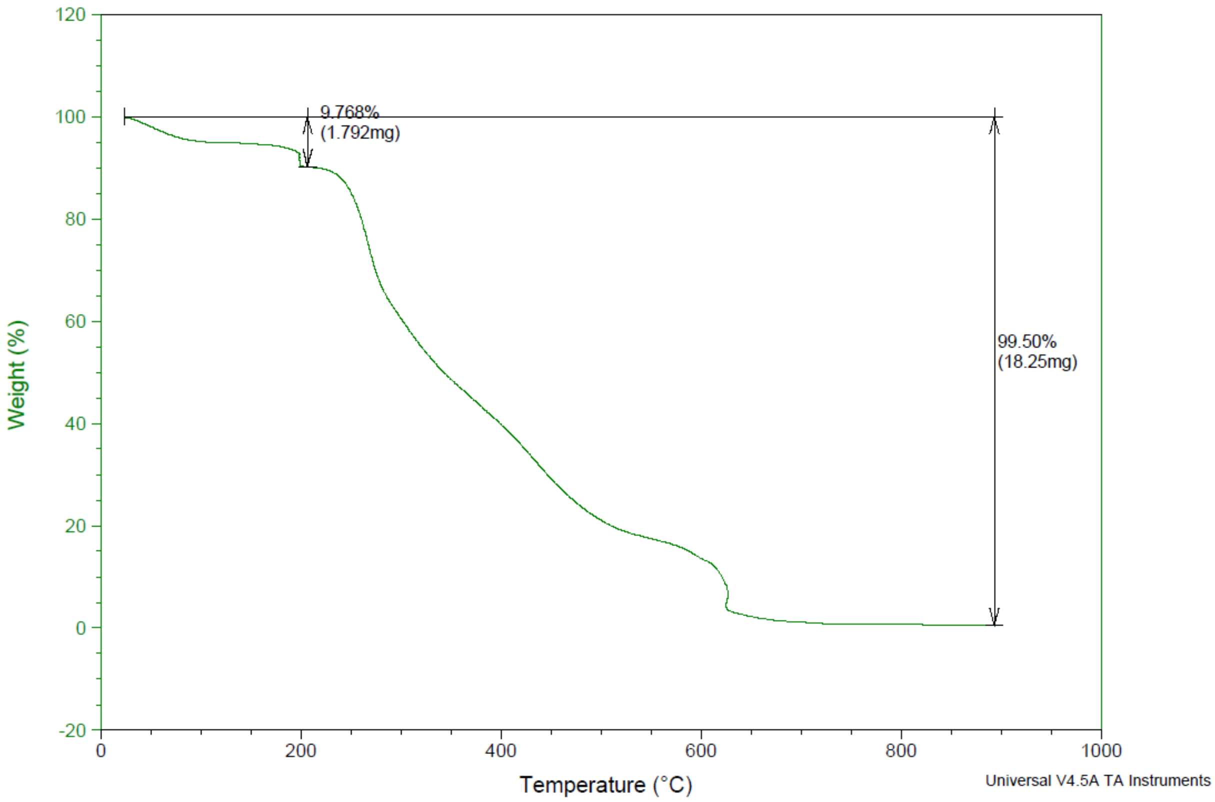


**Figure S11.** TGA thermogram of $COS_{18/35}$.

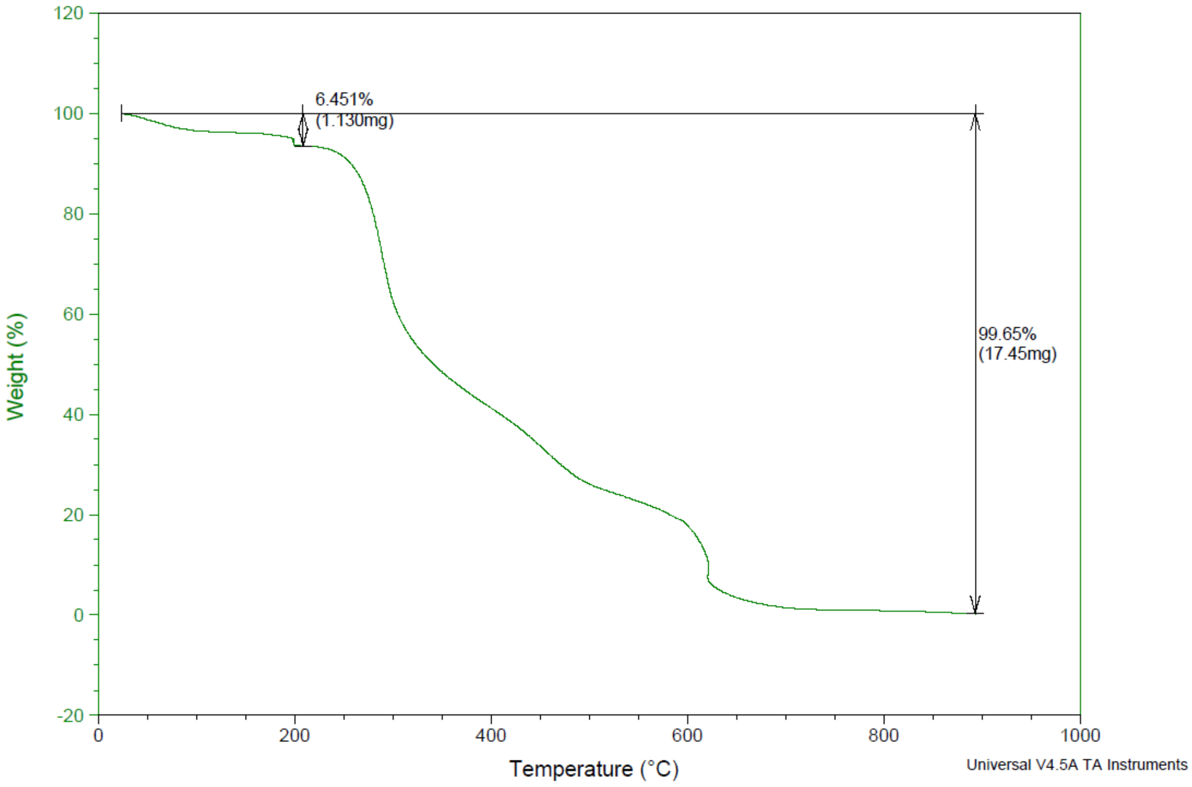


**Figure S12.** TGA thermogram of $COS_{17/51}$.

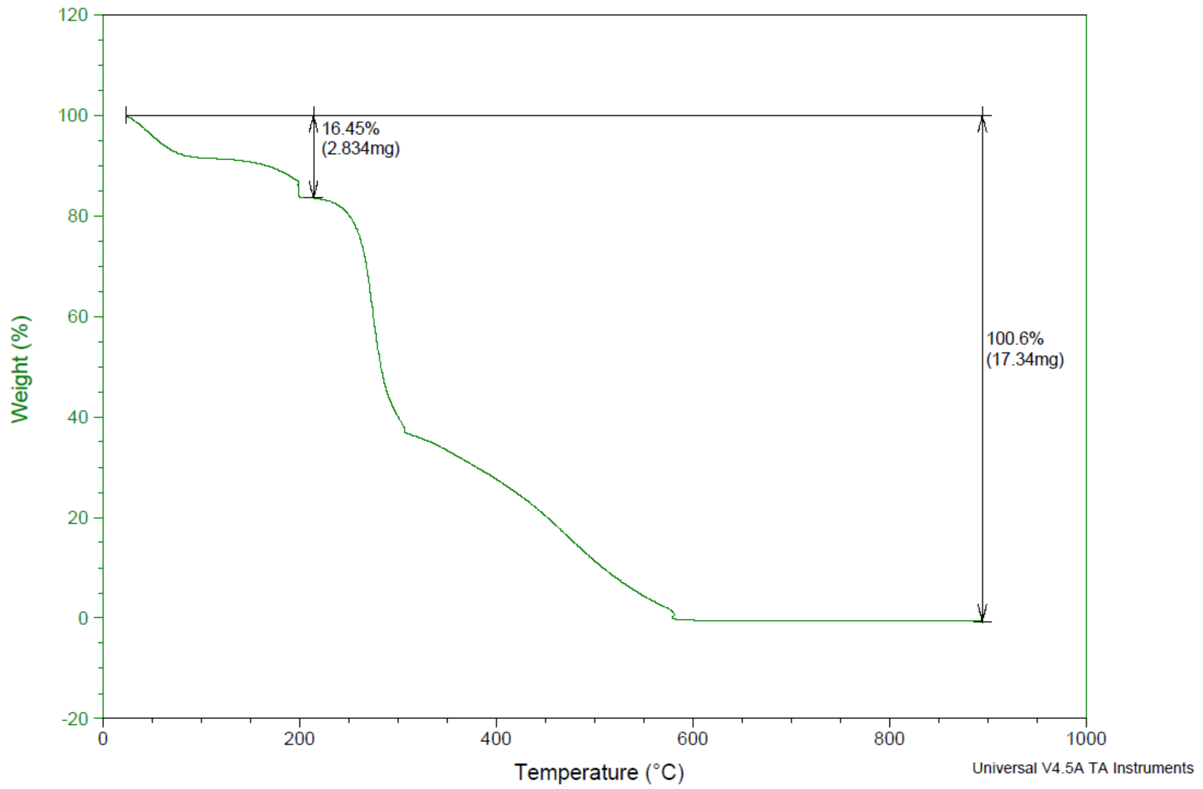


**Figure S13.** TGA thermogram of $COS_{22/52}$.

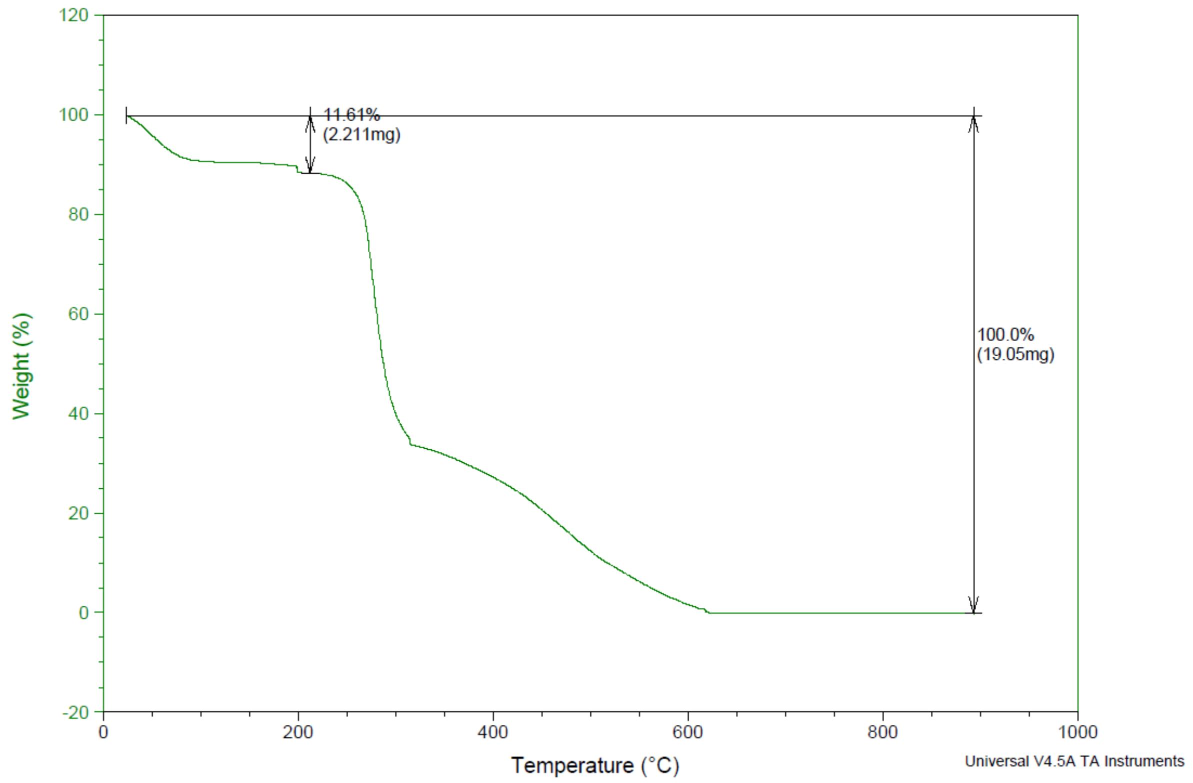


**Figure S14.** TGA thermogram of $COS_{35/57}$.

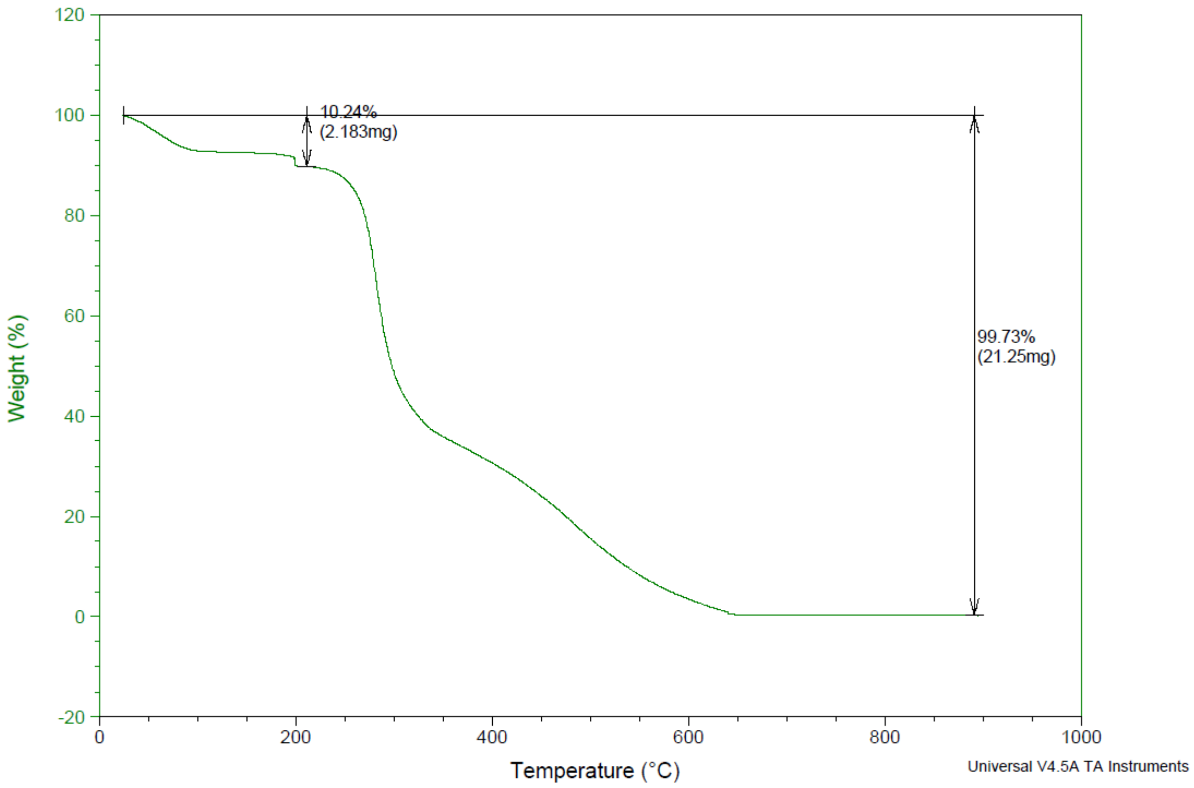


**Figure S15.** TGA thermogram of $CS_{100/49}$.

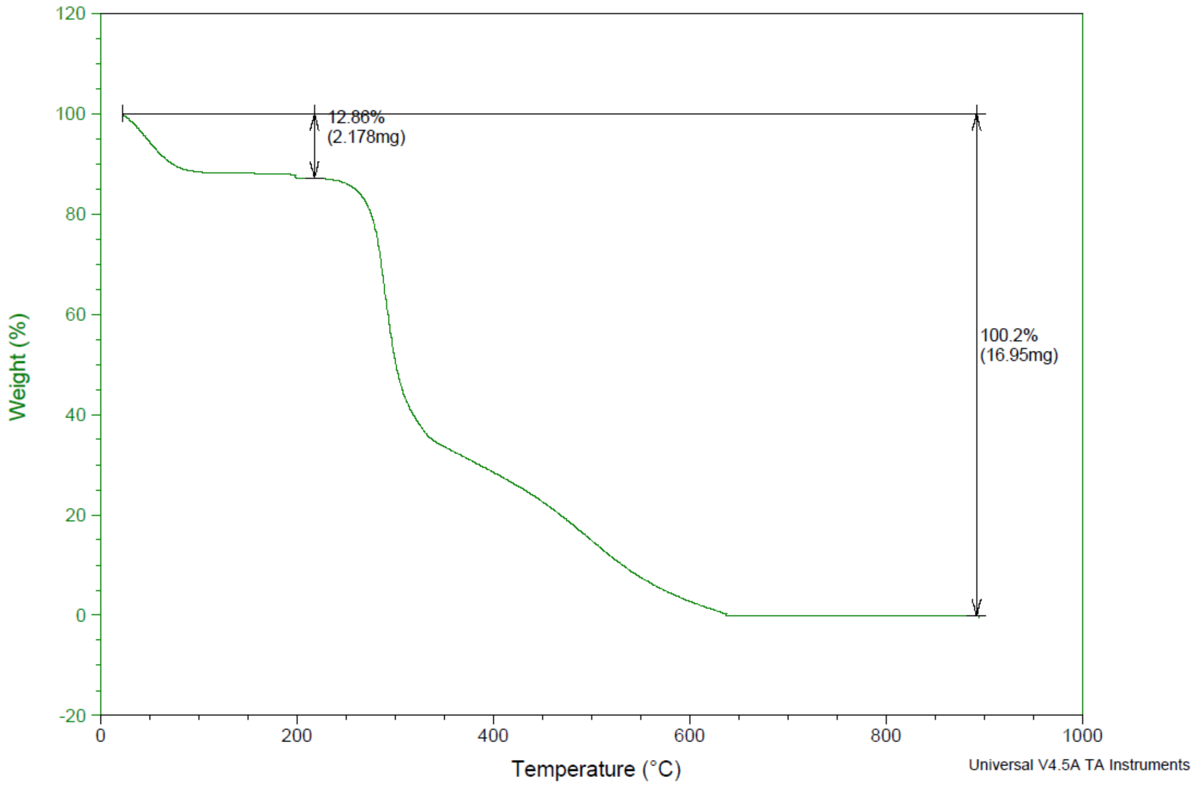


**Figure S16.** TGA thermogram of $CS_{984/50}$.

WYATT TECHNOLOGY

**File Name:** C:\Users\csilvadi.VAS\Desktop\Synthese COS\Analyses_Chitosane\résultats_SEC\RE_ SEC\CSD16 [04avril2023].afe6
**Collection Operator:** LMPB-AF4\aqueux (LMPB-AF4\aqueux (aqueux))
**Processing Operator:** VAS\csilvadi

**Sample:** COS17/1
**Description:**
**Concentration:** 5.200 mg/mL

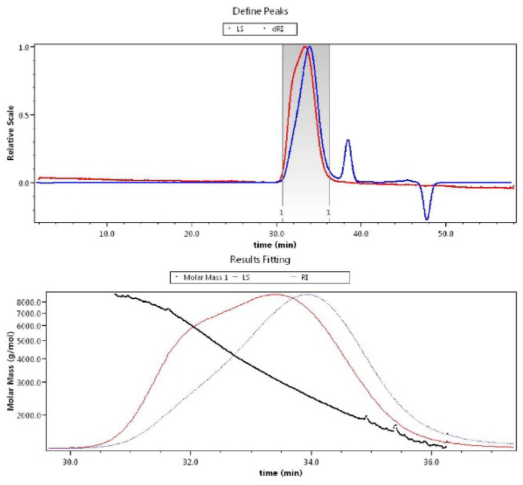


**Configuration**

**Notes:**
Colonnes : TSK6000 et TSK2500, Solvant filtré sur CME 0,1 et échantillon filtré sur CME 0,45

**Concentration Source:** RI
**Flow Rate:** 0.500 mL/min

**Light Scattering Instrument:** HELEOS
**Temperature Control:** yes
**Temperature:** 25.0 °C
**Cell Type:** Fused Silica
**Wavelength:** 664.0 nm
**Calibration Constant:** $3.4807\times10^{-5}$ 1/(V cm)
**QELS Fiber Replaces Detector:** 12 (99.9°)

**RI Instrument:** rEX

**UV Instrument:** UV

**QELS:**
**Use QELS Temperature Probe:** yes
**Model:** Wyatt QELS+

**Solvent:** water
**Description:** Tampon acétate pH=4.8
**Refractive Index:** 1.331
**Viscosity:** 0.894 cP

**Processing**

**Collection Time:** Wednesday April 05, 2023 10:14:03 PM Paris, Madrid (heure dété)
**Processing Time:** Thursday April 20, 2023 09:59:06 AM Paris, Madrid (heure dété)

**Peak settings:**

| | |
|---|---|
| **Peak Name** | Peak 1 |
| **Light Scattering Model** | Zimm |
| **Fit Degree** | 1 |
| **dn/dc (mL/g)** | 0.1980 |
| **A2 (mol mL/g²)** | 0.000 |

**Results Fitting Procedure:**
**Data Fit Model Degree $R^2$ Extrapolation**

**Results**

| Peak Results | Peak 1 |
|---|---|
| **Masses** | |
| Calculated Mass (µg) | 399.01 |
| Mass Recovery (%) | 76.7 |
| Mass Fraction (%) | 100.0 |
| **Molar mass moments (g/mol)** | |
| Mn | $2.677\times10^3$ (±1.447% |
| Mp | $2.536\times10^3$ (±1.229% |
| Mv | n/a |
| Mw | $3.190\times10^3$ (±1.187% |
| Mz | $3.911\times10^3$ (±2.502% |
| **Polydispersity** | |
| Mw/Mn | 1.192 (±1.872%) |
| Mz/Mn | 1.461 (±2.890%) |
| **rms radius moments (nm)** | |
| Rn | 12.1 (±37.1%) |
| Rw | 9.2 (±58.0%) |
| Rz | 5.3 (±159.4%) |

**Figure S17.** Size exclusion chromatogram (0.2M AcOH / 0.15M $AcONH_4$ buffer, pH 4.5) of $COS_{17/1}$.

WYATT TECHNOLOGY

**File Name:** C:\Users\csilvadi.VAS\Desktop\Synthese COS\Analyses_Chitosane\résultats_SEC\RE_ SEC\CSD09dialyse[04avril2023].afe6
**Collection Operator:** LMPB-AF4\aqueux (LMPB-AF4\aqueux (aqueux))
**Processing Operator:** VAS\csilvadi

**Sample:** COS22/0
**Description:**
**Concentration:** 5.300 mg/mL

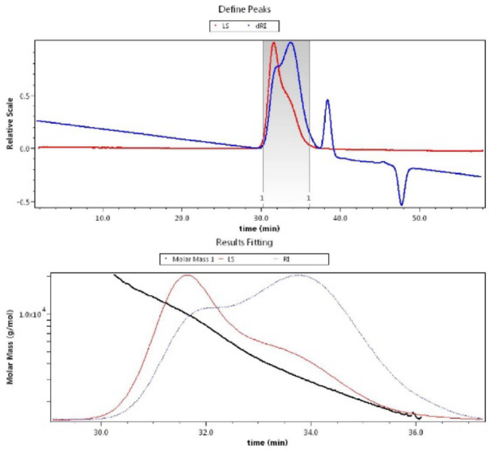


**Configuration**

**Notes:**
Colonnes : TSK6000 et TSK2500, Solvant filtré sur CME 0,1 et échantillon filtré sur CME 0,45

**Concentration Source:** RI
**Flow Rate:** 0.500 mL/min

**Light Scattering Instrument:** HELEOS
**Temperature Control:** yes
**Temperature:** 25.0 °C
**Cell Type:** Fused Silica
**Wavelength:** 664.0 nm
**Calibration Constant:** $3.4807\times10^{-5}$ 1/(V cm)
**QELS Fiber Replaces Detector:** 12 (99.9°)

**RI Instrument:** rEX

**UV Instrument:** UV

**QELS:**
**Use QELS Temperature Probe:** yes
**Model:** Wyatt QELS+

**Solvent:** water
**Description:** Tampon acétate pH=4.8
**Refractive Index:** 1.331
**Viscosity:** 0.894 cP

**Processing**

**Collection Time:** Wednesday April 05, 2023 09:12:14 PM Paris, Madrid (heure dété)
**Processing Time:** Thursday April 20, 2023 09:55:34 AM Paris, Madrid (heure dété)

**Peak settings:**

| | |
|---|---|
| **Peak Name** | Peak 1 |
| **Light Scattering Model** | Zimm |
| **Fit Degree** | 1 |
| **dn/dc (mL/g)** | 0.1980 |
| **A2 (mol mL/g²)** | 0.000 |

**Results Fitting Procedure:**
**Data Fit Model Degree $R^2$ Extrapolation**

**Results**

| Peak Results | Peak 1 |
|---|---|
| **Masses** | |
| Calculated Mass (µg) | 387.33 |
| Mass Recovery (%) | 73.1 |
| Mass Fraction (%) | 100.0 |
| **Molar mass moments (g/mol)** | |
| Mn | $3.532\times10^3$ (±1.106% |
| Mp | $3.141\times10^3$ (±0.823% |
| Mv | n/a |
| Mw | $5.078\times10^3$ (±0.893% |
| Mz | $7.379\times10^3$ (±1.973% |
| **Polydispersity** | |
| Mw/Mn | 1.438 (±1.421%) |
| Mz/Mn | 2.089 (±2.261%) |
| **rms radius moments (nm)** | |
| Rn | 9.2 (±52.2%) |
| Rw | 5.9 (±110.7%) |
| Rz | n/a |

**Figure S18.** Size exclusion chromatogram (0.2M AcOH / 0.15M $AcONH_4$ buffer, pH 4.5) of $COS_{22/0}$.

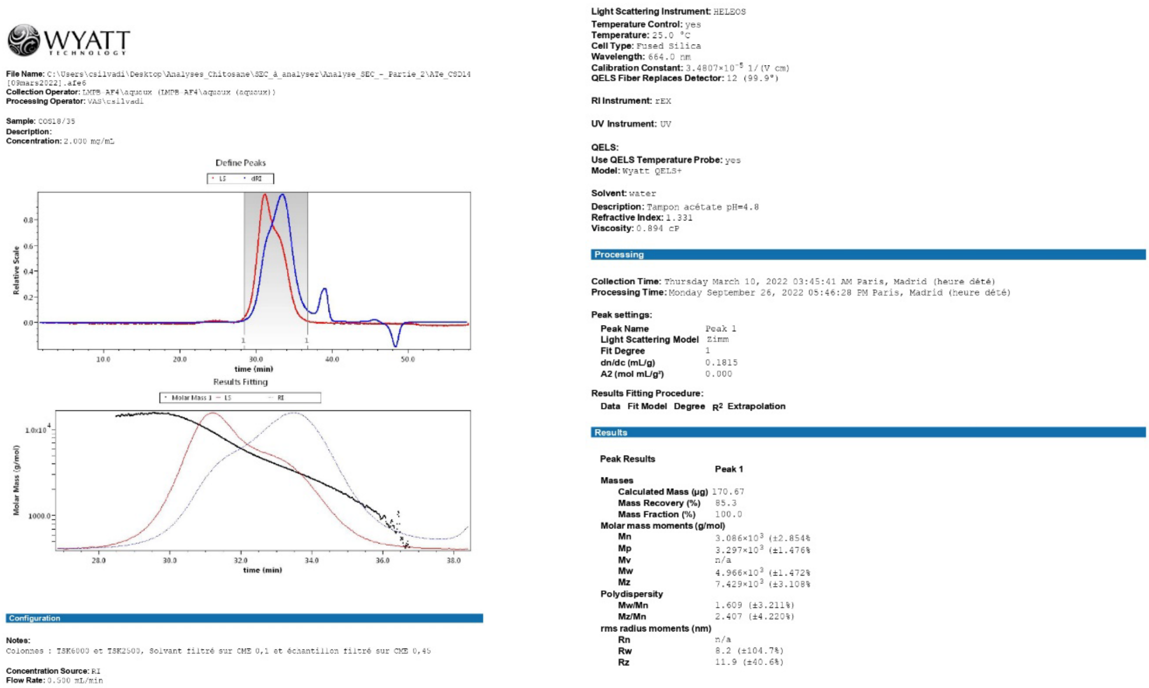


**Figure S19.** Size exclusion chromatogram (0.2M AcOH / 0.15M $AcONH_4$ buffer, pH 4.5) of $COS_{18/35}$.

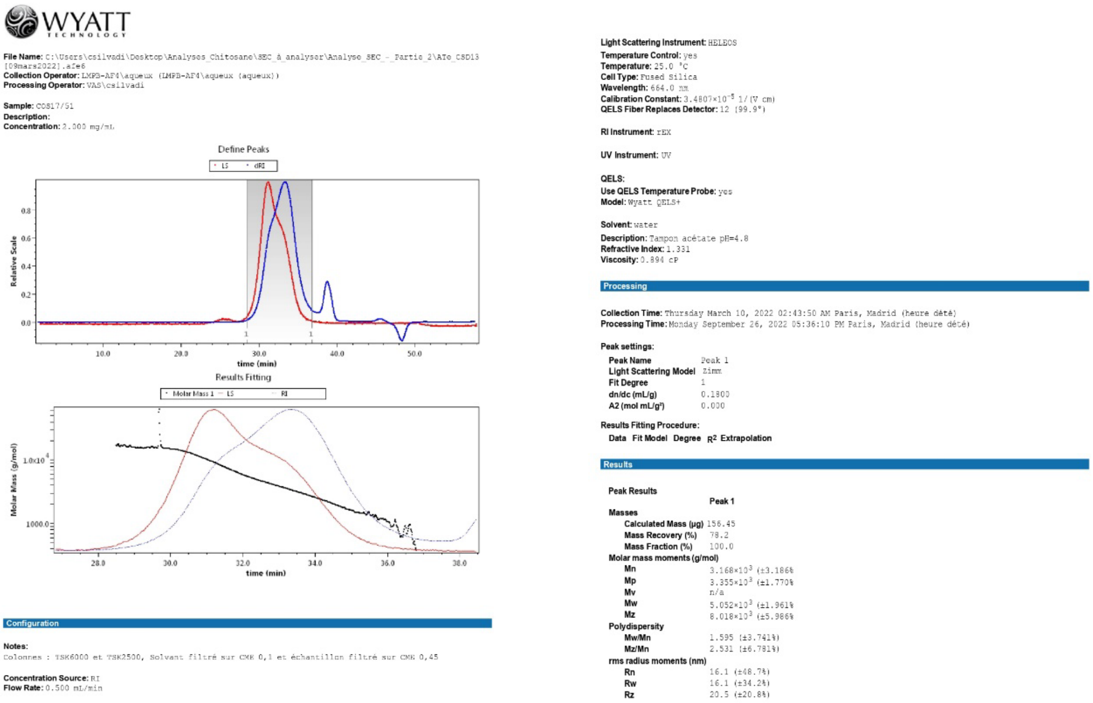


**Figure S20.** Size exclusion chromatogram (0.2M AcOH / 0.15M $AcONH_4$ buffer, pH 4.5) of $COS_{17/51}$.

WYATT TECHNOLOGY

**File Name:** C:\Users\csilvadi\Desktop\Analyses_Chitosane\SEC_à_analyser\Analyse_SEC_-_Partie_1\ATe_CSD10 [09mars2022].afe6
**Collection Operator:** LMPB-AF4\aqueux (LMPB-AF4\aqueux (aqueux))
**Processing Operator:** VAS\csilvadi

**Sample:** COS22/52
**Description:**
**Concentration:** 2.000 mg/mL

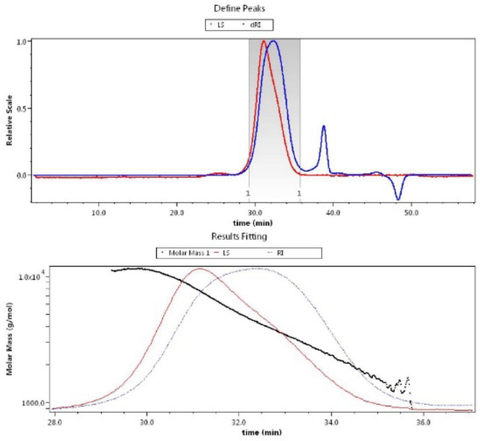


**Configuration**

**Notes:**
Colonnes : TSK6000 et TSK2500, Solvant filtré sur CME 0,1 et échantillon filtré sur CME 0,45

**Concentration Source:** RI
**Flow Rate:** 0.500 mL/min

**Light Scattering Instrument:** HELEOS
**Temperature Control:** yes
**Temperature:** 25.0 °C
**Cell Type:** Fused Silica
**Wavelength:** 664.0 nm
**Calibration Constant:** $3.4807\times10^{-5}$ 1/(V cm)
**QELS Fiber Replaces Detector:** 12 (99.9°)

**RI Instrument:** rEX

**UV Instrument:** UV

**QELS:**
**Use QELS Temperature Probe:** yes
**Model:** Wyatt QELS+

**Solvent:** water
**Description:** Tampon acétate pH=4.8
**Refractive Index:** 1.331
**Viscosity:** 0.894 cP

**Processing**

**Collection Time:** Thursday March 10, 2022 12:40:15 AM Paris, Madrid (heure dété)
**Processing Time:** Monday September 26, 2022 12:20:46 PM Paris, Madrid (heure dété)

**Peak settings:**

| | |
|---|---|
| Peak Name | Peak 1 |
| Light Scattering Model | Zimm |
| Fit Degree | 1 |
| dn/dc (mL/g) | 0.1800 |
| A2 (mol mL/g²) | 0.000 |

**Results Fitting Procedure:**
Data Fit Model Degree $R^2$ Extrapolation

**Results**

**Peak Results**

| | Peak 1 |
|---|---|
| **Masses** | |
| Calculated Mass (µg) | 169.57 |
| Mass Recovery (%) | 84.8 |
| Mass Fraction (%) | 100.0 |
| **Molar mass moments (g/mol)** | |
| Mn | $3.941\times10^{3}$ (±1.788% |
| Mp | $4.351\times10^{3}$ (±1.164% |
| Mv | n/a |
| Mw | $5.165\times10^{3}$ (±1.509% |
| Mz | $6.531\times10^{3}$ (±3.550% |
| **Polydispersity** | |
| Mw/Mn | 1.310 (±2.340%) |
| Mz/Mn | 1.657 (±3.975%) |
| **rms radius moments (nm)** | |
| Rn | n/a |
| Rw | 8.8 (±74.0%) |
| Rz | 12.0 (±39.4%) |

**Figure S21.** Size exclusion chromatogram (0.2M AcOH / 0.15M $AcONH_4$ buffer, pH 4.5) of $COS_{22/52}$.

WYATT TECHNOLOGY

**File Name:** C:\Users\csilvadi\Desktop\Analyses_Chitosane\SEC_à_analyser\Analyse_SEC_-_Partie_2\ATe_CSD15 [09mars2022].afe6
**Collection Operator:** LMPB-AF4\aqueux (LMPB-AF4\aqueux (aqueux))
**Processing Operator:** VAS\csilvadi

**Sample:** COS36/57
**Description:**
**Concentration:** 2.000 mg/mL

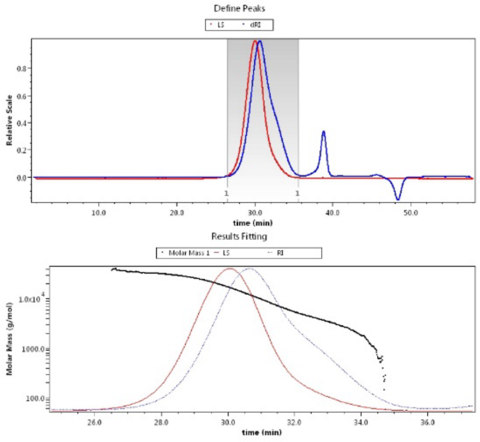


**Configuration**

**Notes:**
Colonnes : TSK6000 et TSK2500, Solvant filtré sur CME 0,1 et échantillon filtré sur CME 0,45

**Concentration Source:** RI
**Flow Rate:** 0.500 mL/min

**Light Scattering Instrument:** HELEOS
**Temperature Control:** yes
**Temperature:** 25.0 °C
**Cell Type:** Fused Silica
**Wavelength:** 664.0 nm
**Calibration Constant:** $3.4807\times10^{-5}$ 1/(V cm)
**QELS Fiber Replaces Detector:** 12 (99.9°)

**RI Instrument:** rEX

**UV Instrument:** UV

**QELS:**
**Use QELS Temperature Probe:** yes
**Model:** Wyatt QELS+

**Solvent:** water
**Description:** Tampon acétate pH=4.8
**Refractive Index:** 1.331
**Viscosity:** 0.894 cP

**Processing**

**Collection Time:** Thursday March 10, 2022 04:47:31 AM Paris, Madrid (heure dété)
**Processing Time:** Monday September 26, 2022 05:49:59 PM Paris, Madrid (heure dété)

**Peak settings:**

| | |
|---|---|
| Peak Name | Peak 1 |
| Light Scattering Model | Zimm |
| Fit Degree | 1 |
| dn/dc (mL/g) | 0.1800 |
| A2 (mol mL/g²) | 0.000 |

**Results Fitting Procedure:**
Data Fit Model Degree $R^2$ Extrapolation

**Results**

**Peak Results**

| | Peak 1 |
|---|---|
| **Masses** | |
| Calculated Mass (µg) | 180.47 |
| Mass Recovery (%) | 90.2 |
| Mass Fraction (%) | 100.0 |
| **Molar mass moments (g/mol)** | |
| Mn | $6.600\times10^{3}$ (±3.419% |
| Mp | $1.216\times10^{4}$ (±0.984% |
| Mv | n/a |
| Mw | $1.205\times10^{4}$ (±1.085% |
| Mz | $1.718\times10^{4}$ (±2.439% |
| **Polydispersity** | |
| Mw/Mn | 1.825 (±3.587%) |
| Mz/Mn | 2.603 (±4.200%) |
| **rms radius moments (nm)** | |
| Rn | n/a |
| Rw | 8.1 (±72.9%) |
| Rz | 10.9 (±34.8%) |

**Figure S22.** Size exclusion chromatogram (0.2M AcOH / 0.15M $AcONH_4$ buffer, pH 4.5) of $COS_{36/57}$.

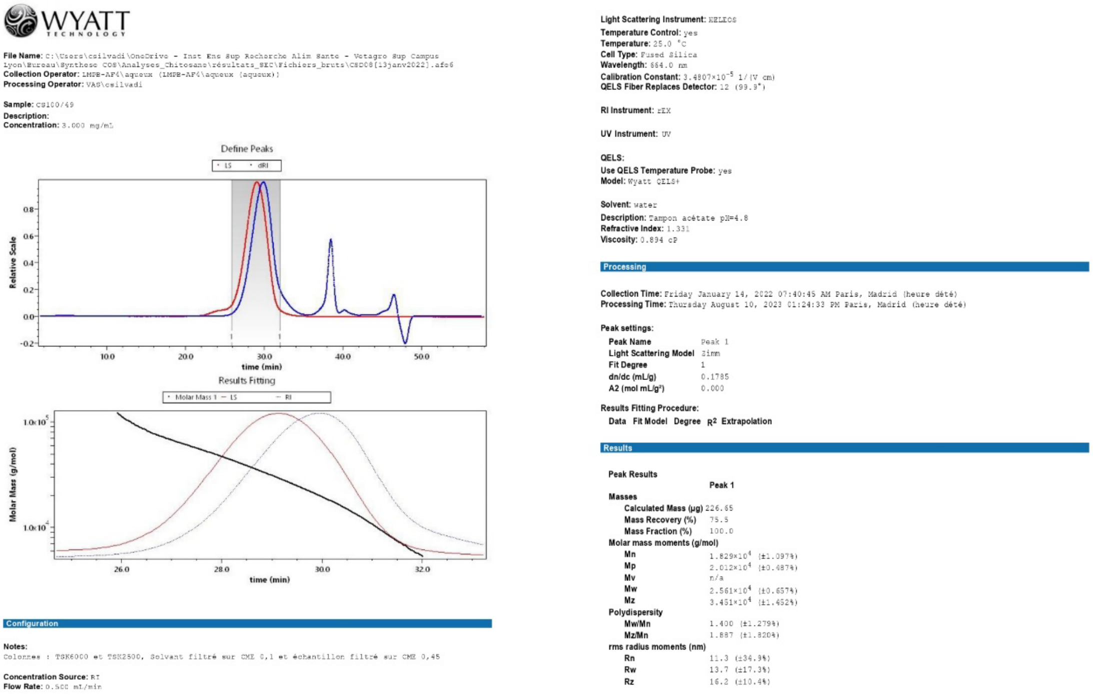


**Figure S23**. Size exclusion chromatogram (0.2M AcOH / 0.15M $AcONH_4$ buffer, pH 4.5) of $CS_{100/49}$.

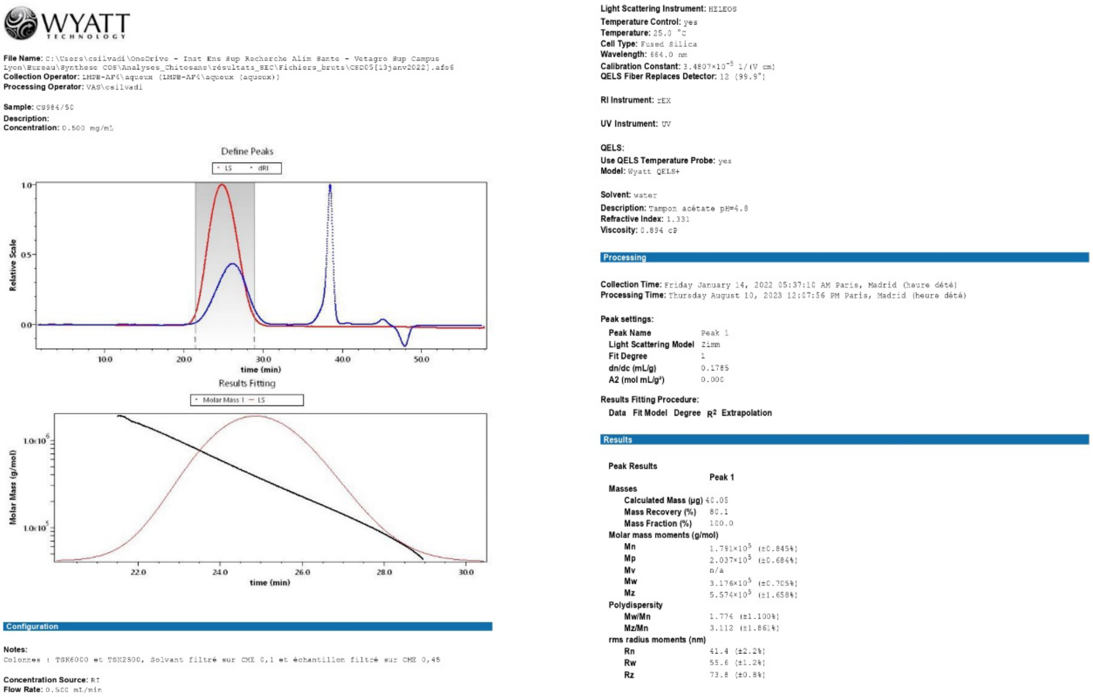


**Figure 24.** Size exclusion chromatogram (0.2M AcOH / 0.15M $AcONH_4$ buffer, pH 4.5) of $CS_{984/50}$.
